# The Immune Epitope Database and Analysis Resource Program 2003–2018: reflections and outlook

**DOI:** 10.1007/s00251-019-01137-6

**Published:** 2019-11-25

**Authors:** Sheridan Martini, Morten Nielsen, Bjoern Peters, Alessandro Sette

**Affiliations:** 1 Division of Vaccine Discovery, La Jolla Institute for Immunology, 9420 Athena Circle, La Jolla, CA 92037 USA; 2 Department Health Technology, Technical University of Denmark, Kgs. Lyngby, Denmark; 3 Instituto de Investigaciones Biotecnológicas, Universidad Nacional de San Martín, Buenos Aires, Argentina; 4 Department of Medicine, University of California San Diego, La Jolla, CA USA

**Keywords:** T cell, B cell, Antibody, Epitope, Database, Epitope prediction tool

## Abstract

The Immune Epitope Database and Analysis Resource (IEDB) contains information related to antibodies and T cells across an expansive scope of research fields (infectious diseases, allergy, autoimmunity, and transplantation). Capture and representation of the data to reflect growing scientific standards and techniques have required continual refinement of our rigorous curation and query and reporting processes beginning with the automated classification of over 28 million PubMed abstracts, and resulting in easily searchable data from over 20,000 published manuscripts. Data related to MHC binding and elution, nonpeptidics, natural processing, receptors, and 3D structure is first captured through manual curation and subsequently maintained through recuration to reflect evolving scientific standards. Upon promotion to the free, public database, users can query and export records of specific relevance via the online web portal which undergoes iterative development to best enable efficient data access. In parallel, the companion Analysis Resource site hosts a variety of tools that assist in the bioinformatic analyses of epitopes and related structures, which can be applied to IEDB-derived and independent datasets alike. Available tools are classified into two categories: analysis and prediction. Analysis tools include epitope clustering, sequence conservancy, and more, while prediction tools cover T and B cell epitope binding, immunogenicity, and TCR/BCR structures. In addition to these tools, benchmarking servers which allow for unbiased performance comparison are also offered. In order to expand and support the user-base of both the database and Analysis Resource, the research team actively engages in community outreach through publication of ongoing work, conference attendance and presentations, hosting of user workshops, and the provision of online help. This review provides a description of the IEDB database infrastructure, curation and recuration processes, query and reporting capabilities, the Analysis Resource, and our Community Outreach efforts, including assessment of the impact of the IEDB across the research community.

## Overview and introduction

Our initial focus in the 2003–2011 period was to design and render operational the Immune Epitope Database (IEDB) and associated Analysis Resource (IEDB-AR) (Peters et al. 2005a; Peters et al. 2005b; Vita et al. 2010; Zhang et al. 2008). In the second quarter of 2011, the IEDB reached the key milestone of being up-to-date with curation of published immune epitope data within its scope (Salimi et al. 2012). Then, and now, it remains a priority that we continuously optimize processes, since the number of epitopes/year steadily increases. Due to the unprecedented amount of data accumulated, in the 2012–present period, we introduced significant enhancements in the database structure, usability, and query capacity (Vita et al. 2019; Vita et al. 2015). Likewise, the performance and breadth of the existing tools within the Analysis Resource were improved while designing altogether new classes of tools (Dhanda et al. 2019). These activities were designed to fulfill the ongoing aim of facilitating the analysis, compilation, and display of the large amount of data available and to support epitope prediction and analysis based on data and sequences provided by the users.

The LJI team was awarded support for the IEDB for a new period, spanning years 2019–2025. Throughout this period, our vision will be to meet the challenge of data growth and complexity, and to offer the best available bioinformatics tools to the epitope community. The new cycle of work is associated with distinctive opportunities and challenges. We will continue to provide a one-stop resource to catalog and analyze immunological data; including B cell and T cell recognition and MHC binding data, and also the exponentially growing amounts of data related to natural ligands and epitope-specific BCR/TCRs. The vision includes parallel growth of the tools and algorithms available to the community.

Throughout these efforts, we will continue building the IEDB in support of the broad movement that creates and brings full utilization to community-based ontologies and data standards. As such, a key component of both design and outreach activities is to connect the epitope data in the IEDB with other knowledge resources such as the BRCs, ImmPort, IMGT, PDB, UniProt, and NCBI. Realizing this vision also requires meeting significant challenges in terms of infrastructure. The original IEDB was designed in 2003, and it dealt with a data landscape of much lesser volume and complexity. In the last 5 years alone, although the number of published references per year remains fairly constant, the average number of epitopes published per reference has increased 12-fold, and the number of unique visitors to the IEDB websites has doubled. These trends are expected to continue and would put a static IEDB design under pressure. Keeping up with exponential increases in data content and user-base will require constant enhancement of IEDB systems and operations.

## Database infrastructure

**Fig. 1:**
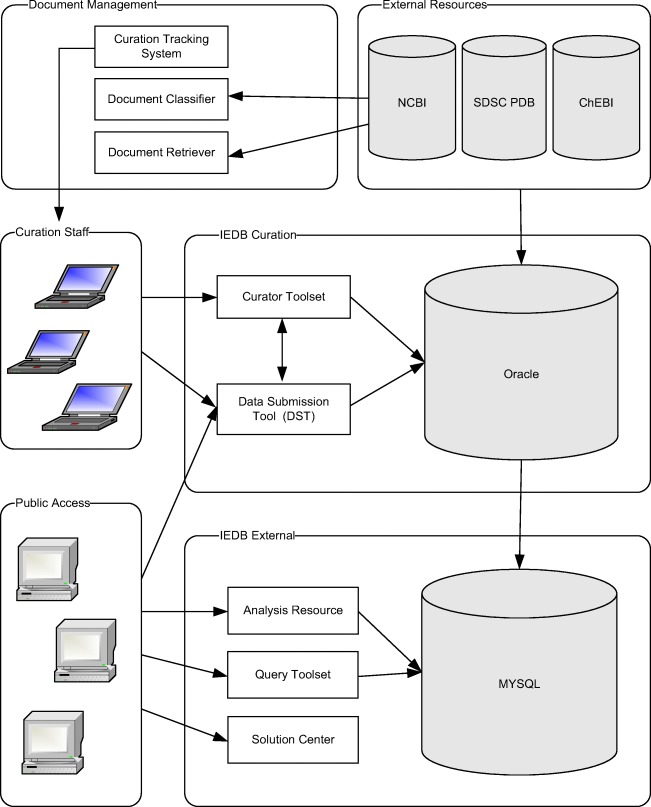
Diagram of database structure

Continuously maintaining and enhancing the database-associated infrastructure is key to accommodate increases in the volume of data contained in the database, increases in the number of users, and future technological advancements. The infrastructure is based on three separate database systems, namely External, Curation, and Submission (Fig. 1). In the External system, epitope data from multiple sources can be queried and analyzed by external users. The associated Curation system allows IEDB staff to capture immune epitopes and accompanying biological information from the scientific literature. Finally, the Submission system allows investigators from the broader research community to send their epitope data and accompanying biological information to the IEDB.

Currently, all database and application servers are run on Virtual Machines (VMs) that are strategically deployed across multiple hardware platforms in different locations, including cloud environments. This is well coordinated, and all of the virtual machines are cloned from a validated ancestor VM to ensure they are consistent and reliable when deployed. This approach allows for rapid recreation of clean instances should there be corruption, and a safety net for system administrators and programmers as they interact with these systems. Although many of the technologies were harmonized and security-patched over time, it is anticipated that in the coming years, several areas will benefit from major updates and/or complete replacements.

The IT architecture used for the IEDB needs to accommodate a large number of differences in the types of applications and users which have variable hardware and/or network configurations and are located at both domestic and international sites. Accordingly, efforts are directed to ensure that our architecture is capable of accommodating this variability among users worldwide. To this end, we set up a VMware vSphere computing cluster hosting virtualized machines at LJI (sometimes referred to as a “private cloud”). With all systems running on the VMware vSphere cluster, hardware and networking components standardized to efficiently assign resources when and where needs arise.

## Web portal

The IEDB web portal (www.iedb.org) is the central access point for external users to the IEDB data and tools. The present website encompasses several interfaces to query and browse curated data; tools to predict, analyze, and visualize epitopes; user support in the form of specific tutorials and help desk; and other resources such as downloads of the full database; a news section with reports, manuscripts, and compendia; and a list of links to related resources. Maintenance of these systems is routine, but parallel activities aim to continually enhance the web portal to optimize the value of the IEDB for its user community.

A thorough and formalized process is in place to identify, prioritize, and implement enhancements in the IEDB web portal. Indeed, the IEDB portal was completely redesigned based on input received from usability studies and user feedback, to reduce the content in the first page and focus the user on the most frequently used features. Our formalized process identifies potential enhancements based on feedback from the IEDB team itself, interactions with existing users and the wider scientific community, and feedback from the NIAID. On an annual basis, we review suggestions, provide a preliminary feasibility assessment based on the time and resources necessary for implementation, then for selected projects demonstrate and test prototypes before release.

To ensure usability of the web portal, we continuously evaluate interfaces for practical flow and usability with the scientific community. It is of particular importance that the user interface (UI) is clear and intuitive and it is not uncommon for scientific websites to focus on providing a large number of features while neglecting the UI. A common blind spot is found when evaluation is limited to only those directly involved in developing functionality interface elements; because in fact, those who are best-suited to judge if a feature is easy to use are those who are less familiar with its development. To avoid this pitfall, we routinely conduct tests of new functionality by outside users from the scientific community. These recommendations were a major component of the IEDB 2.0 and 3.0 redesigns (Vita et al. 2015; Vita et al. 2010).

We engage experts in the field of epitope identification, but also bioinformatics and experimentalists across areas in immunology. These experts can be identified, for example, by querying authors most prolific in publishing papers in a specific subject or engaging representatives of other related and relevant NIAID programs. Ultimately, the broader scientific community is the best and most important judge of the adequacy and effectiveness of the IEDB, so this feedback on the scientific value and accuracy of the interfaces is of crucial importance.

Based on site and conference surveys, junior scientists (those at the postdoctoral level) are the largest user group of the IEDB. Accordingly, we engage postdoctoral fellows from LJI and other local research institutions, presenting volunteers with UIs (either existing ones or new prototypes) and monitoring them as we ask to solve a scientific question. We have also found it beneficial to perform reviews of the entire website by professional, external usability consultants focused on ensuring that the navigation structure and UI design is consistent with best practices for human-computer interaction.

**Fig. 2:**
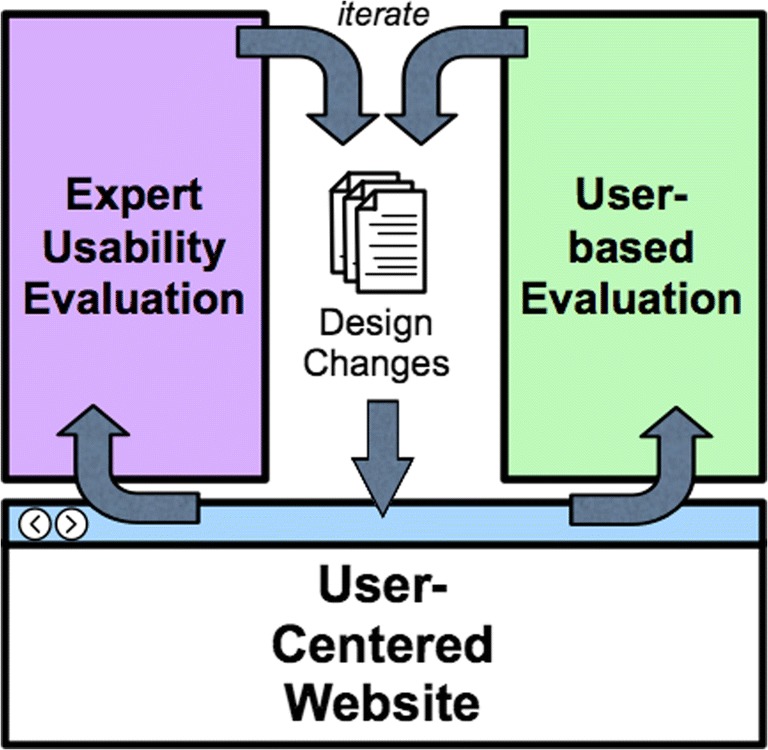
Process to improve website usability

Convergence of each described evaluation technique drives the IEDB development feedback cycle depicted in Fig. 2. Through constant collection and assessment of this information, the IEDB team is able to focus efforts on core concepts most desired by the community and to implement new features following basic UI paradigms which ultimately streamline data accessibility. Specific examples of the IEDB UI development are detailed in the “Query and reporting” section below.

## Maintaining the data currently in the database through recuration

Data recuration is the process by which previously curated and public data records are updated to improve retrieval and/or clarify content. This process is critical to optimize the IEDB as it is reflective of scientific progress and priorities driven continuously by community needs. A continuous stream of suggestions for recuration arises from outreach activities (user feedback, interactions with other NIAID supported programs), errors identified by validation rules, and the integration of ontologies. A number of external ontologies are integrated into the IEDB’s curation and search interfaces as “finder” applications including the Organism finder utilizing the National Center for Biotechnology Information (NCBI) Taxonomy (Coordinators 2018), the Molecule finder utilizing UniProt reference proteomes (The UniProt 2018) and Chemical Entities of Biological Interest (ChEBI) (Hastings et al. 2013), the Assay finder that uses the Ontology for Biomedical Investigations (OBI) (Bandrowski et al. 2016), the MHC Restriction Finder utilizing the MHC Restriction Ontology (MRO) (Vita et al. 2016), and the Geolocation finder that uses the Gazetteer Ontology (GO) (Ashburner 2015). For each finder, we annually review the hierarchy to uncover curation errors and test each tree for functional synonyms.

For example, when mapping IEDB disease states to the Disease Ontology (DO) (Schriml et al. 2012), we identified curation errors regarding the relationship between the disease and the infectious agent curated as causing that disease. For example, the disease “Hepatitis C infection” cannot be caused by the “Hepatitis B” virus agent. Similarly, “*Plasmodium falciparum*” malaria cannot be caused by “*Plasmodium vivax*” agent. Errors such as these were corrected and will be prevented going forward by the implementation of validation rules that rely upon the logical definitions found in DO.

We rely on automated validation as a key process to identify data in need of recuration. Several layers of automated validation are applied throughout the curation process, thus preemptively addressing potential recuration needs and issues. This computer-based validation was first introduced in 2008, and since undergone constant and significant expansion. Currently, the validation file contains 253 separate rules. Separately, we have also begun to make use of logical axioms defined by ontologies to enhance our validation system. We expect that we will continue to expand these validation rules, and to specifically modify them to reflect the changes in the database structure or ontology that may be introduced as a result of the proposed work and continued evolution of the IEDB data and structure.

Since the database in under constant scrutiny and feedback is continuously received through a variety of channels, recuration tasks are collated throughout the year and then reviewed to determine need and feasibility on an annual basis. To prioritize and select suggestions for action, we first consolidate the list of change requests, eliminating duplications and overlap; conduct a cost-benefit analysis; and finally nominate the highest priority tasks for immediate action, while flagging some lower priority tasks for possible action if resources become available.

## Populating the database with data from scientific literature

Populating the database with new data from the literature relies on an established processes and procedures for epitope curation, which have been optimized over 16 years of maintaining the IEDB. During this time, the IEDB has become the predominant resource of epitope information containing data on over 2 million experiments from over 20,000 papers. The overall process is based on identifying and classifying the suitability of journal articles, curating each of the selected papers, peer-reviewing the curated records, and finally promoting approved records to the external IEDB site. In addition to processing newly published, high priority in-scope papers, a “backlog” of outstanding papers is simultaneously monitored. The backlog consists of published literature which is in-scope (i.e., containing epitope-related information) but does not fall within our high priority curation categories of infectious disease, autoimmunity, allergy, or transplantation. In 2011, the IEDB team hit a landmark goals of completing the historical curation of all papers published prior to establishment of the IEDB itself. Since then, the number of outstanding papers has varied between ~ 100 and ~ 1000 papers; the fluctuation is mostly associated with new categories of papers being prioritized and added to the curation pipeline, causing an influx to the total number of papers. Our team tracks the number of new papers being published and curated per period of time. The effort allocation is designed so that the rate of papers curated exceeds the rate of newly introduced papers, allowing us to reduce the backlog and effectively tackle new paper categories over time.

### Identifying curatable journal articles

**Fig. 3:**
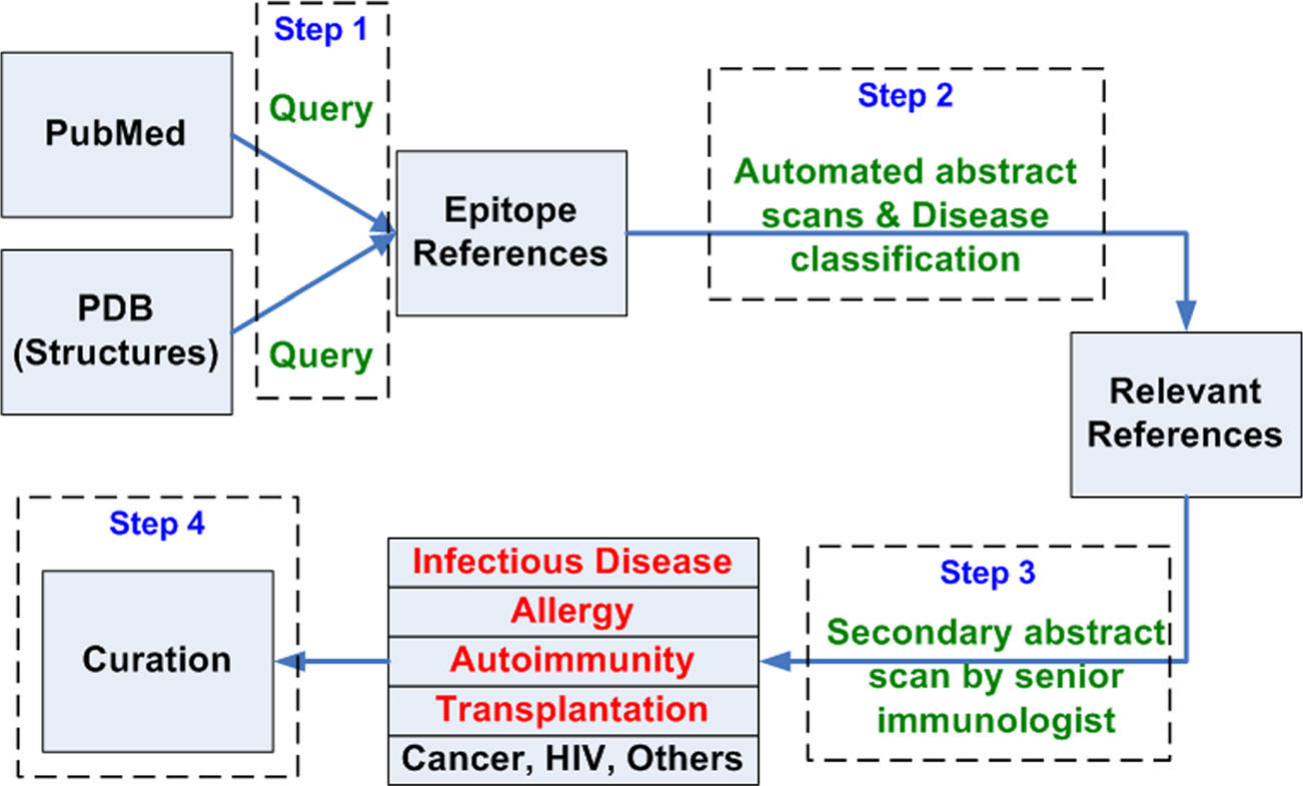
Workflow for identifying curatable journal articles

A formalized rigorous process is utilized to identify the journal articles that will be curated, based on the combined use of PubMed and PDB queries (Fig. 3, step 1), automated text classifiers and categorizers (Fig. 3, step 2), and manual inspection of records by senior immunologists (Fig. 3, step 3) (Fleri et al. 2017). While the total number of missed references cannot be known, the number of missed references identified as a result of user and expert feedback is approximately 10 per year out of an annual average of 858 curated references, corresponding to 1.2%. These data suggest that the PubMed query is capable of capturing the vast majority of relevant references. References that were missed are examined as to at which step they were discarded, and used to update the query and classification procedure.

We utilize a general query based on abstracts to identify potentially curatable articles (Seymour et al. 2011; Wang et al. 2007). Utilizing this type of continuously updated query since program inception in 2003, we have identified over 229,000 abstracts of potential relevance, spanning the entire history of papers cataloged in PubMed. Upon each execution, the query identifies new PubMed records, which correspond to newly published reports or papers from earlier years that have since been added to the PubMed repository. While this query process has been developed and optimized over a long period of time, we found that the query’s recall rate can drop when new fields of expertise are targeted by the curation efforts or new technologies are introduced in the scientific literature, leading to new keywords and query modifications introduced as a result.

The abstract of each reference identified by the PubMed query is further analyzed to determine whether the reference meets the IEDB inclusion criteria. The specific criteria are described in more detail in our online curation manual and previous publications (Fleri et al. 2017; Vita et al. 2008; Vita et al. 2006). To minimize human inspection of the large number of abstracts, and to maintain low and acceptable error rates, we established and validated a semi-automated process in which iteratively trained document classifiers are used to eliminate papers with high probability of being uncuratable (Seymour et al. 2011; Wang et al. 2007). The probability threshold associated with the automated classifier was intentionally set high, so that no more than a 5% false negative rate would be tolerated. Senior immunologists on the IEDB team then manually inspect each of the abstracts deemed curatable by the classifier. We retrain the classifier annually utilizing the results of manual curation.

### Curating journal articles

Once a paper has been selected for curation, a process involving curation of the experimental data and peer review of the curated record is utilized to ensure correctness, completeness, and consistency (Vita et al. 2008). Throughout curation, the epitope-related data are entered into the database utilizing an internal web interface specifically developed to enhance curation speed, accuracy, and consistency. Many fields use controlled vocabularies that are made available to the curators by a variety of finders facilitating the selection of appropriate field values. For example, the Organism finder for antigens and hosts is based on the NCBI taxonomy, and the Assay finder utilizes entries in OBI. In addition to the finders, many of the fields have drop-down menus that the curators use to select from a limited number of allowable values. The design of the system has undergone several iterations and continues to be improved upon and expanded utilizing feedback from the curation team.

Quality control is ensured by several system-enforced mechanisms throughout the curation process. First, as data are being entered, the website applies embedded validation rules (as mentioned above) ensuring entered data match the field type and are properly formatted. Second, each curation is peer-reviewed by an independent curator for accuracy and adherence to the IEDB curation guidelines. This peer review process is iterative, and a reference will not be released to external users until both the curator and the reviewer are satisfied with the quality of the curated data.

Each paper is tracked in our Curation Tracking System, indicating the paper’s status in the curation pipeline: assigned to a curator, in curation, initial curation complete, in review, and finalized for promotion to the database. The timeline for each one of these steps is also recorded systematically, and when analyzed, helps the curation team identify process bottlenecks and consider possible solutions to improve throughput.

### Populating the database with naturally processed MHC ligand data

In physiological conditions, MHC molecules are mostly found with their peptide-binding site occupied by naturally processed (NP) peptides derived from proteins expressed within the cell, or acquired from the extracellular milieu. Their capture is of significant interest to characterize possible epitopes recognized in the context of autoimmunity and cancer, and increasingly as a powerful approach to discern potential candidates for epitope identification studies. The ability to compare NP data with MHC binding data allows us to gain insight into antigen processing and potentially develop new predictive algorithms.

Currently, NP data represent over 400,000 total ligands derived from more than 500 submitted and published papers, spanning the time-period of 1990 through 2019. While the vast majority of these NPs were “conventional” peptides, over 20,000 ligands with post-translational modifications were also reported (Vaughan et al. 2017). Not surprisingly, most of the NP ligand data have been defined from humans with rodent NP data far less abundant. Our process for reference classification already identifies and categorizes NP epitope papers. These references are currently processed, utilizing established procedures, by curators specifically familiar with NP data curation.

Parallel efforts in the mass spectrometry (MS) community have led to database developments capturing primary data, such as selected reaction monitoring (SRM), data-dependent acquisition (DDA), and data-independent acquisition (DIA) (Caron et al. 2015). We do not envision that the IEDB will act as a repository of this primary data, which will continue to be stored in the various resources specifically dedicated to NP data. We envision that the IEDB will specialize in making available data on the ultimate epitope recognized, using link-in and link-out functionalities, the associated metadata, and thus enable the integrated analysis of data from different studies and resources.

Integrated approaches are key to ensure interoperability of data repositories. To this end, the Human Immuno-Peptidome Project (HIPP), associated with the Human Proteomic Organization (HUPO), provides a collaborative and integrative conduit for immunopeptidomic data storage and analysis. In parallel, we will continue to capture NP data from the various peptidome repositories, such as SWATHAtlas and, develop links between the IEDB and the various peptidome repositories so users can access relevant information.

### Maintaining and further enhancing a central source of BCR and TCR repertoire data

From its initiation, the scope of the IEDB has been to capture experiments defining epitope-specific immune responses. Recent years have seen technological advances in the generation of BCR and TCR sequence data. Cutting-edge research is now defining the universe of TCR and BCRs present in the general population, under steady-state conditions (non-diseased) and as a result of perturbation, such as diseases, infections, vaccination, and aging (Rubelt et al. 2017). Most importantly in this context, the BCR and TCR repertoires associated with recognition of specific antigens are starting to be defined and understood. Parallel recent research suggests that knowledge of BCR/TCR sequences might actually be utilized to predict the specific epitopes recognized by these effector cells (Glanville et al. 2017). These advances have the potential to revolutionize our understanding of adaptive immunity and lead to novel diagnostic, therapeutic, and vaccine applications. As these receptor data are reported differently than epitope data by themselves, we adjusted our curation efforts and are continuously curating and recurating records related to epitope-specific antibodies and TCRs with known sequences. We have introduced appropriate modifications to the IEDB curation processes and underlying systems (ontologies, validation checks and so on) to support this new data type.

The processes implemented to curate these sequences have been described in detail in a recent review (Mahajan et al. 2018). In short, to identify which journal articles contain epitope-specific receptor information, we expanded our query for keywords (e.g., “TCR repertoire,” “CDR3 sequence”), to retrieve additional papers. To further enhance our query, we also examined several databases dedicated to antigen-specific receptors such as Atlas DB, Harvard DB, DTU, VDJ DB, and AdBio, to identify additional keywords in articles included in those databases that were missing from the IEDB. After identifying the relevant references, we determined what information should be captured beyond the description of the epitope in the IEDB, by community standards (Breden et al. 2017; Rubelt et al. 2017), which recommend submitting the raw sequence information of receptor repertoires to Sequence Read Archive (SRA) (Leinonen et al. 2011), submitting the assembled receptor repertoires to dedicated repositories of such data that follow AIRR recommendations (such as VDJserver, iReceptor), and linking to the IEDB to identify specific epitopes recognized by receptors.

### The IEDB-3D curation

IEDB-3D is the 3D structural component of IEDB (Ponomarenko et al. 2011). As of January 2019, there are 2555 B cell, 276 T cell, and 1075 MHC curated 3D structural assays. The curated data include receptor, epitope, and antigen sequences, and other structure data such as curated and calculated residue contacts between antigen and receptor included. Newly published manuscripts containing this data are identified through a biweekly PDB query targeting crystal structures that include an antibody, TCR, or MHC molecule based on analysis of the sequence of the crystalized protein chains. If epitope-paratope contact information is provided in the references, it is captured in the database as “curated contact” information. In addition, the contact information is also always calculated in all cases and included in the record as “calculated contacts.” We also distinguish CDR regions from full receptor chain sequences based on the IMGT numbering scheme (Lefranc et al. 2003) using ANARCI (Dunbar and Deane 2016), which enables searching for e.g., antibodies with a specific heavy chain CDR3 sequence identified in either crystallography or repertoire sequencing data. Since the 3D data can be used for developing and validating epitope prediction algorithms, the dataset has been made available as a separate, continually updated download that provides a simplified data structure aimed at structural biologists.

### The evolution of the curation needs and targets throughout the IEDB life span

**Fig. 4:**
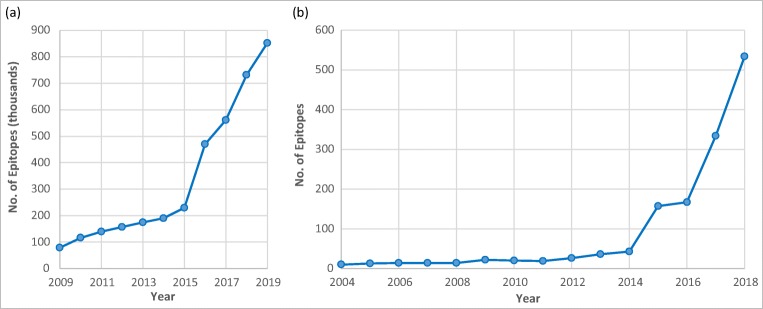
Epitope counts annually within the IEDB (**a**) and averages per curated paper (**b**)

Over the last ten years, the IEDB epitope content (in terms of total number of epitopes) has increased approximately 10-fold or ~ 20%/year, with a sharp rise since 2015 (Fig. 4a). Despite the fact that the IEDB is now in “maintenance mode” for the epitope categories originally envisioned to be in scope, data growth shows no sign of abating and is, in fact, accelerating. To identify the cause of this accelerated growth, we performed quantitative analyses, on papers curated 2004–2018.

This phenomenon resulted from a progressive increase in the number of epitopes per paper (Fig. 4b), despite the number of new epitope papers appearing in the literature slightly decreasing over time. Keeping up with this growth in addition to tackling new categories of epitope papers necessitated a continued effort to increase a curator’s capacity to process data. Our analysis showed that curator output increased approximately eight-fold over the last decade, and will likely need to increase further. Improving curation efficiency and throughput remains a key focus of the IEDB efforts.

## The Data Submission Tool

**Fig. 5:**
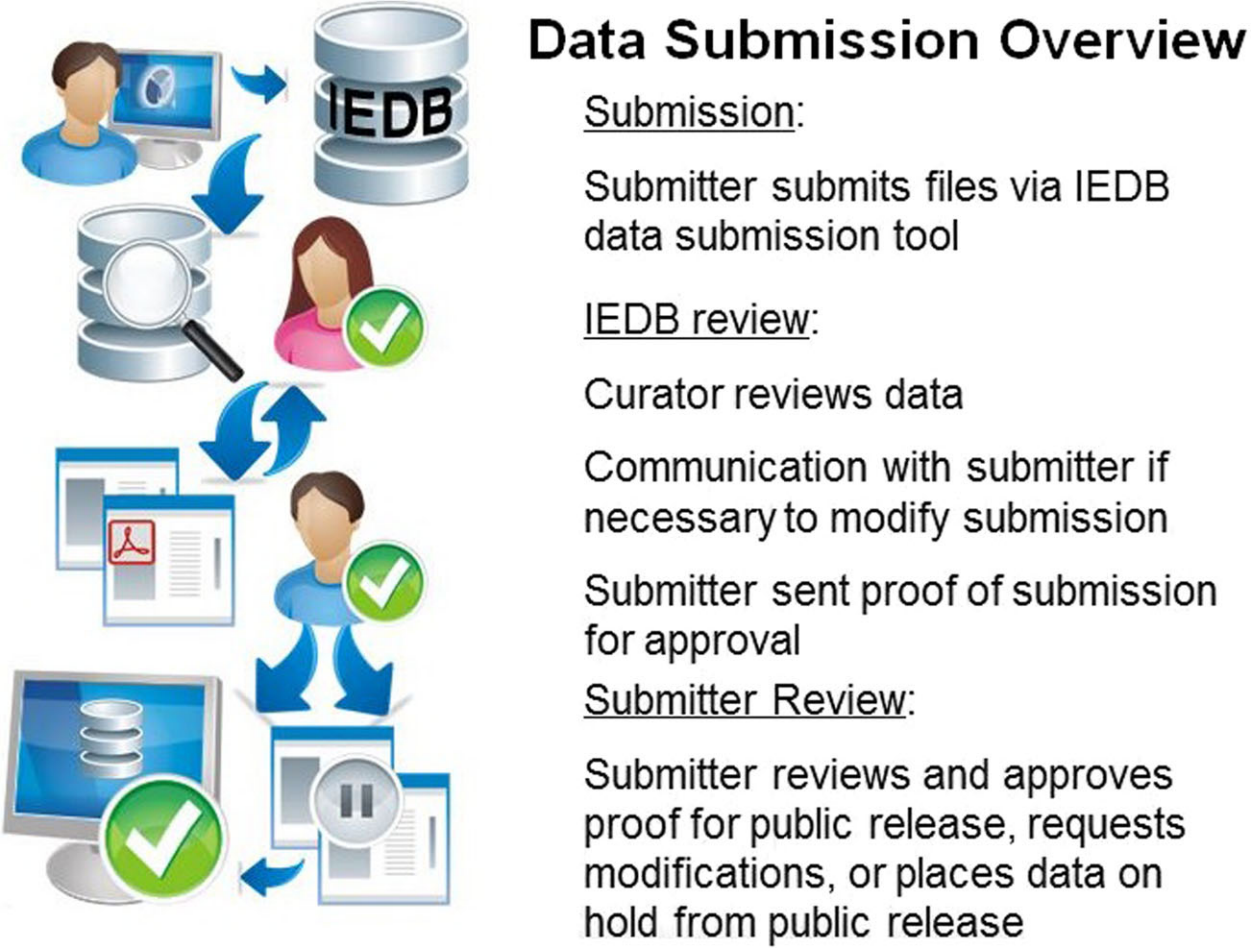
Workflow of investigator initiated data submissions

The database is also populated through direct investigator submissions. Support for these submission requests has resulted in the generation of the Data Submission Tool (DST) which provides multiple channels for data submission: Extensible Markup Language (XML) file submission, a spreadsheet Microsoft Excel file submission, and online wizard-assisted submissions. Regardless of submission method, all approaches follow the same workflow depicted in Fig. 5. Data submitters first contact the IEDB website, are issued an account, and can then access the DST interface, where template files and explicit directions (as well as help) can be obtained. After dataset validation, and once both the IEDB staff and the submitter are satisfied, the data are approved for public release on the IEDB; however, timing of release is ultimately at the submitter’s discretion since often investigators prefer to wait until publications are at least in press.

On average, the IEDB team receives 35 data submission requests containing around 40,000 epitopes/peptides per year. A major source of data has been the NIH-funded epitope discovery contracts. Of 353,468 epitopes submitted electronically, the IEDB had processed 191,149 epitopes submitted by investigators from 22 epitope discovery contracts as of 31 December 2018.

Since the DST was introduced at the start of 2009, 98% of submission requests have been made via spreadsheets. This makes apparent that the IEDB data submission community strongly prefers utilizing spreadsheets. We will thus focus on the spreadsheet submission going forward and will incorporate aspects of the other systems, namely stringency of the XML submission and user-friendly guidance of the wizard system into the spreadsheet submission itself.

The current spreadsheet templates for T cell, linear B cell, discontinuous B cell, MHC binding, MHC ligand elution, and non-peptidic data have been in place for several years and have had slight revisions, as needed, based primarily on modifications to the data schema. We are currently developing new versions of the templates to accommodate BCR/TCR sequence data and make modifications to the MHC ligand elution template including enhancing data validation and error reporting.

## Query and reporting capabilities enable access to data throughout the IEDB

The IEDB content continuously evolves in terms of volume and information type; this evolution is paralleled by a continuous enlargement in the number and types of users. We regularly modify and optimize query and reporting strategies to balance this growth. To accomplish this goal, we gather feedback regarding the usability and desired enhancements of the IEDB UI. In the past, surveys via SurveyMonkey were deployed as a link on the IEDB home page and sent to members of the epitope discovery groups, subscribers to the IEDB Solutions Center, people who had inquired and/or signed up for the User Workshops, and users who had submitted help requests. User observation sessions were also conducted in order to collect usability metrics and feedback. Comments were combined with all requests received from the help desk as additional input and revealed a clear overall message that the website needed to be easier to use, and that most users wanted to perform simple queries, which needed to be immediately obvious how to perform.

**Fig. 6:**
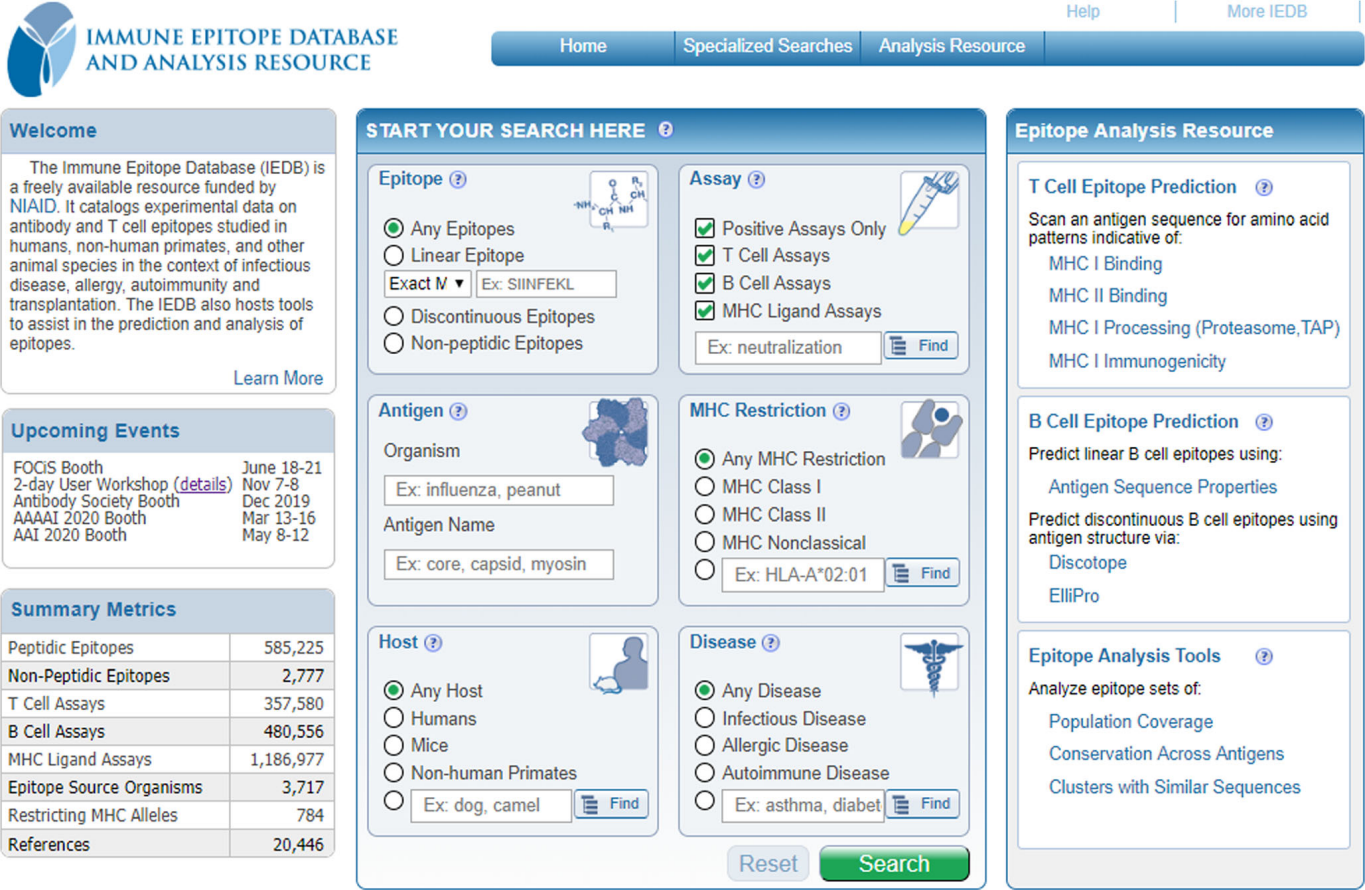
IEDB 3.0 Homepage

Once the main user needs were identified, they were scrutinized by usability experts who provided feedback focused on the placement of control elements, color patterns, font sizes, use of icons, and other features that their research showed to improve the ability of users to effectively navigate web pages. Following multiple iterations, a final prototype was completed and demonstrated to a representative group of users for further feedback. The final result of this process was the IEDB 3.0 homepage redesign (Vita et al. 2015) shown in Fig. 6, which moved the main query functionality onto the home page and focused it on high interest elements. Different sets of query results are then presented on a web page with different tabs that match the query parameters. Additional controls on the result page allow to further narrow down the results. This tab-based presentation of results matches a common web page interface paradigm that users are already familiar with. One of the most common examples is travel websites, which present flights, hotels, and car rentals in response to a query for travel to a certain city. This redesign was a major success, with a significant drop in the rate of users that visited the IEDB home page but leave without further action because they could not understand how to proceed (i.e., the “bounce rate,” as defined by Google Analytics).

Another significant component of the IEDB 3.0 redesign was the addition of “finder applications” that assist users in specifying antigens, MHC alleles, assays, source and host species, and diseases. These applications combine the use of autocomplete functionality in textboxes that are synonym-aware, with tree-based views of term taxonomies, clearly depicting the hierarchical relationships among terms sourced from community ontologies.

### Implementing a dedicated search interface for TCR/BCR sequences

To develop a targeted search interface, we introduced a receptor specific search pane on the results page (Mahajan et al. 2018). This search pane allows users to search for all epitopes having antibodies or TCRs with known sequence information or to further narrow those results by specific receptor type (e.g., alpha beta TCR) or chain type (e.g., light chain). Additionally, this search pane allows one to search by full-length or CDR sequence and utilizes a BLAST match functionality, including limiting sequence identity levels (90–60% or substring matches). This pane also includes the option to search for all epitopes for which there is a known 3D structure of its binding to an antibody or TCR.

Additionally, a new receptor results tab was introduced, next to the pre-existing epitope, antigen, assays, and references results tabs. This tab shows the receptor ID, the species in which the receptor was generated in, the type (alpha/beta TCR, etc.) and, if available, the CDR3 sequences of chains 1 and 2. To display the detailed results associated with each record, we implemented a new page specifically for this purpose. Receptor information such as ID, name, receptor type, and chain 1/2 gene usage and sequence information is included. In addition, information regarding the epitope(s) associated with each receptor is provided, including sequence, epitope ID, antigen, and organism of origin, along with the specific immunogenic assays reported for each epitope.

### Complying with FAIR principles through ontology and external links

The FAIR principles (Findability, Accessibility, Interoperability, and Reusability) were introduced in 2016 to serve as essential principles which should drive the design of data repositories to best optimize the usefulness of their data holdings (Wilkinson et al. 2016). Since the functional purpose of the IEDB is to support human users querying through the website’s graphical browser interface, the relevance of these principles to our mission was immediately realized.

Our user community predominantly consists of experimental scientists, so most effort thus far has gone into making the query and reporting interfaces accessible without any advanced computational skills. At the same time, and in partnership with those in the database community, significant effort has been made towards making the IEDB data more computable. This is done to utilize automated inferences for data validation, to enable advanced query interfaces (Vita et al. 2013), and with full intent that we improve links between the IEDB and other knowledge repositories (Peters and Sette 2007).

Immediately following the release of these standards, we conducted a review of the IEDB’s compliance to assess the degree of compliance, to identify areas of non-compliance, and simultaneously to explore how the FAIR principles might be adjusted and fine-tuned to facilitate applications benefitting the general community (Vita et al. 2018; Vita et al. 2017). As result, we established a list of fields and terms to be added to the IEDB, and redesigned the export file. Also, to further integrate the IEDB data with BD2K/FAIR resources, we added Internationalized Resource Identifiers (IRIs) to exports, added provenance to data, and are standardizing data locations.

### Analysis of performed queries to optimize interface designs

A key goal of the IEDB query interface design is that the queries users want to perform most frequently are also the easiest to perform. We periodically review the actual queries performed to determine if the fields made most prominently available should be updated to reflect any changes in user needs. Specifically, we analyze all queries performed either directly from the home page or using additional filter capabilities in the results page or queries performed from the detailed search pages, by tracking how many times each field is used. Our goal is to ensure that 95% of all queries performed on the site can be formulated via the home page search interface directly, rather than requiring additional search fields, and that 99% of all queries are possible when including the additional filter capabilities on the results page. To ensure continued achievement of these goals, we analyze the actual queries performed on a yearly basis identifying any areas requiring adjustment.

For example, reviewing results of a query analysis over 21 weeks and 46,400 queries, we found that 90% of queries were possible on the home page and 96% of all queries were possible on the results-filter page. We next analyzed, in more detail, home page usage, in terms of distribution of field usage by section. The epitope and antigen sections were most used (29% and 20%, respectively), followed by assay and host (16% and 12%), and then MHC restriction and disease (7% and 4%). In terms of specific fields, the most commonly used field was structure type (peptidic, non-peptidic, etc.) with a count of 21,047 uses. In 2nd place, we found source organism (16,421), 3rd was linear sequence/value (16,008), and 4th was host (15,875). Other commonly used fields were no B cell assays (5th; count of 9275), epitope source antigen (6th; 8980), MHC class (7th; 8423), filter by epitope (8th; 7667), no MHC assays (9th; 7508), disease (10th; 4895), and no T cell assays (11th; 4313). These results were encouraging, since they indicated that 98.6% of utilized fields are present on the home page. They also indicated that additional work would be required to ensure that 95% of all queries could be executed on the home page, since only 90% of all queries were possible from there. We next rectified this by moving additional fields onto the home page, such as the ability to search for specific assay types. In doing this, we accomplished our goal of allowing 95% of all queries performed on the site be possible directly via the home page search interface.

### Implementing the reference epitope reporting functionality

**Fig. 7:**
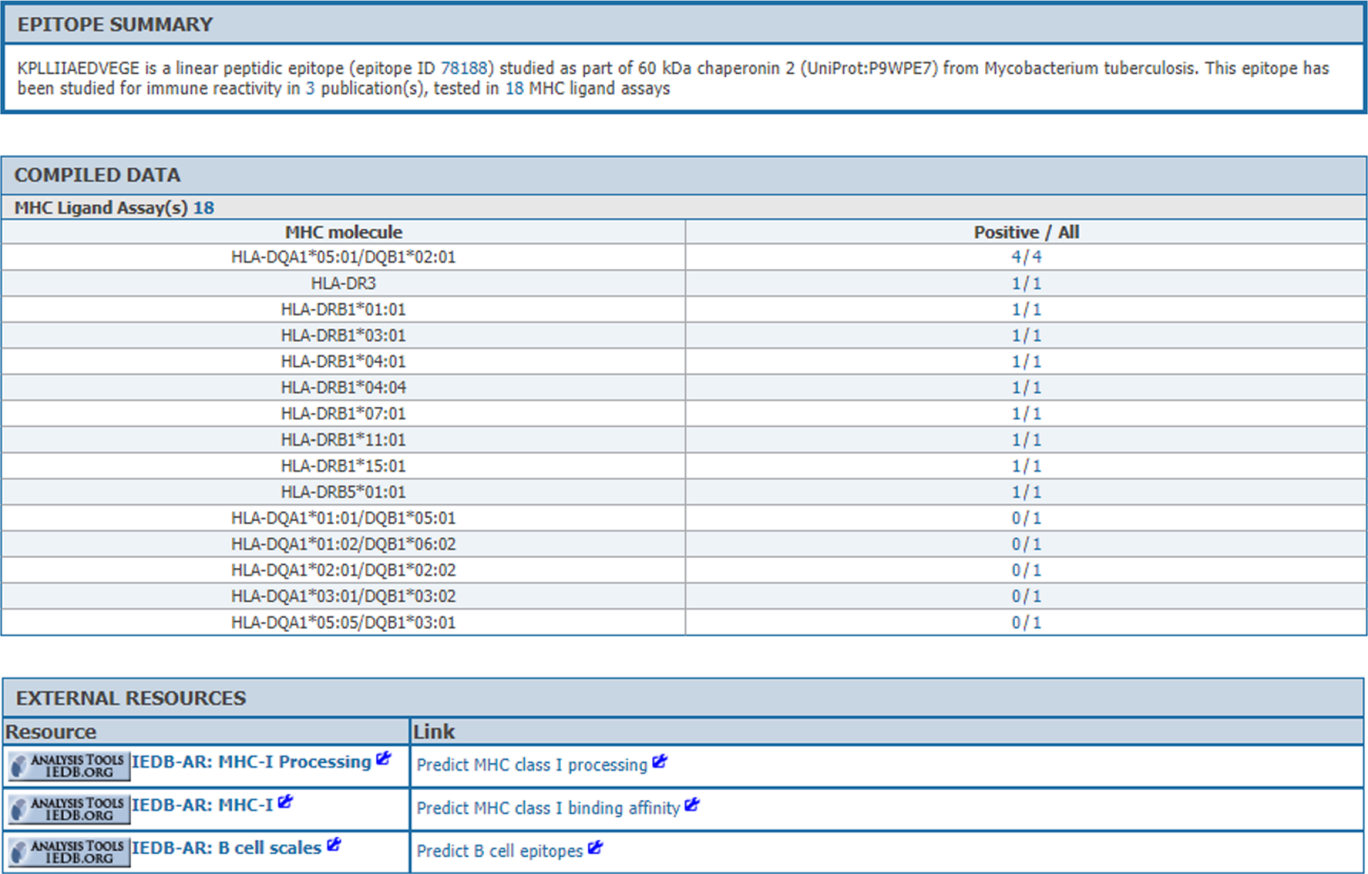
Example Reference Epitope page

A common user request was to summarize results available rather than providing tables with many rows that report individual experimental data points. To address this, we began implementation of the “Reference Epitope” concept. The goal of this function is to summarize experimental results automatically using computational algorithms in order to report information in a succinct manner. An initial version of such algorithms was tested and recently released on the public website (Fig. 7). The reference epitope starts with a textual summary of the data for a given epitope, such as, “KPLLIIAEDVEGE is a linear peptidic epitope (epitope ID 78188) studied as part of 60 kDa chaperonin 2 (UniProt:P9WPE7) from Mycobacterium tuberculosis. This epitope has been studied for immune reactivity in 3 publication(s), tested in 18 MHC ligand assays.” The words underlined in the example provide live links that the user can use to directly access the information. As intended, this presentation strategy provides a high-level summary and immediately below, the user can view tables that capture the most frequently requested information available in the IEDB (such as MHC binding data, listing the alleles for which data are available, and the number of positive assays/total).

We are in the process of generating similar views for B cell assays, MHC restriction, protective/neutralization data, and other categories for which relevant data are available. In our vision, the development of the reference epitope represents an important new functionality and uniquely exploits the value afforded by our ontology work. We envision that these pages summarizing the entirety of data available will also be a useful link-in/link-out target for other resources (e.g., PubChem, WikiData).

## The IEDB Analysis Resource

The LJI team effort builds upon its very successful record of accomplishment in making the IEDB Analysis Resource a major resource for analyzing and predicting T and B cell epitopes (Dhanda et al. 2019; Kim et al. 2012; Zhang et al. 2008). The general objectives in managing the Analysis Resource are to maintain and enhance existing tools, to introduce new tools, and to provide unbiased benchmark evaluations across all publically available prediction methods.

As requests for new tools and upgrades to existing tools began to be collected, we realized a distinct conflict between making tools available to the community quickly versus guaranteeing that what is made available will provide accurate and stable results over time. Thus, a new LABS classification was introduced in 2016. Tools under AR Labs are experimental and are considered to be beta versions, not quite ready for production yet, for which we anticipate that new versions with fundamental changes could be developed without advance user consultation. This differentiates from CORE tools where we will announce non-incremental updates in advance and obtain feedback from the user community before implementation.

The tools provided in the analysis resource fall into two primary categories—prediction tools and analysis tools. Prediction tools extrapolate beyond data held in the database and can be used to predict epitopes in protein sequences or predict properties of known epitopes, such as their MHC binding affinity. Analysis tools help extract and interpret data contained in the database by identifying characteristics of existing datasets.

### Maintaining existing epitope analysis tools

Presently, four analysis tools have been implemented at the CORE level. First, the Population Coverage tool (Bui et al. 2006) calculates the fraction of individuals predicted to have the capacity to respond to a given set of T cell epitopes with known MHC restrictions. This calculation is made on the basis of HLA genotypic frequencies assuming non-linkage disequilibrium between HLA loci. Second, the Epitope Conservancy Analysis tool (Bui et al. 2007) calculates the degree of conservancy of a peptide within a given set of protein sequences at adjustable levels of sequence identity. The degree of conservation is defined as the fraction of protein sequences containing the peptide at a given identity level. Third, the Epitope Cluster Analysis tool (Dhanda et al. 2018b) groups user-provided peptides into clusters based on sequence identity. A cluster is defined as a group of sequences that have a similarity greater than the minimum threshold specified. As of March 2019, this tool will be upgraded to a new version to address user requests for different modes of clustering. Fourth, a web page describing Computational Methods for Mapping Mimotopes to Protein Antigens. Rather than providing a specific tool, this web page provides information on how to search the IEDB for mimotopes and how to utilize external, publicly available tools to map them to proteins, including examples of a mimotope dataset and the mapping results.

Over the years, we established robust procedures to ensure tools remain functional. Our experience has allowed us to learn of possible failure points and to implement procedures that prevent issues from reoccurring. Specifically, all tools in the IEDB-AR CORE are continuously monitored for availability and functionality by Zabbix, which probes if the tool provides expected responses. In addition to this automated monitoring, we continuously receive user feedback and bug reports, which can identify problems that are not covered by the automated monitoring and occur, for example, only with a specific combination of user input. As problems are identified, we follow industry standard software development practices to address them: a ticket is filed with the problem description and assigned to an IEDB tool developer. The problem will then, if possible, be recreated in a test case, which is integrated into our test suite to ensure that if the same problem reoccurs, it will be detected before it gets to our production systems. The tool code will be updated and tested to determine if the problem is fixed following our standard testing cascade.

### Developing new tools

In addition to the four analysis tools described above, two epitope analysis tools have recently been added which are classified as LABS tools. The novel RATE (Restrictor Analysis Tool for Epitopes) application (Paul et al. 2015) can infer HLA restriction for a set of epitopes based on patterns of T cell responses in HLA-typed subjects. The RATE tool takes two data files, one containing the alleles expressed by the subjects and the other containing the response to the peptides in these subjects. The tool calculates the odds ratio of an HLA allele being associated with positive responses for a given peptide and estimates its significance using Fisher’s exact test. The ImmunomeBrowser was also recently added under LABS. This tool enables visualization of the known immune response to a specific antigen. Specifically, it provides the immune reactivity in terms of response frequency (RF) and the number of subjects tested/responded and/or number of independent assays performed along the length of reference protein. Based on a similar feature originally implemented in the results page of the database section of the IEDB, this standalone version extends usability to predicted epitopes and propriety epitopes or non-IEDB data (Dhanda et al. 2018c). The standalone version maps user provided peptide sets and associated response data to a user-provided protein reference sequence. This now allows the user to analyze and visualize immunodominant regions within their own dataset.

Our approach is to couple all new tool releases with a peer-reviewed journal publication detailing the rationale, background, and methodology applied for tool development as well as provide examples of usage and validation of expected outcomes. Such a publication serves not only to alert potential users of its existence, but the peer review process also provides external input and review of our efforts and forces a higher quality standard for the documentation.

New tools added to the IEDB-AR do not necessarily have to be developed from scratch but can also be adopted from external tool developers interested in hosting tools. For example, the MHC-NP tool (Giguere et al. 2013) performed very well in a benchmark of NP and presented peptides but was not made available to the public so our team contacted the developers to determine if they were agreeable to hosting the tool on the IEDB-AR. In cases like this, it cannot be expected that the external tool developers will follow the IEDB coding guidelines. Instead, we work with the external developer to provide a Python-wrapper that encapsulates the tool, which can then go through the same testing procedures described above. The potential downside of this approach is that problems that are part of the external code inside of the wrapper might not be fixable by our team. However, in practice, all problems encountered can be detected and independently fixed at the level of the wrapper code, or they can be fixed with the help of the external developers.

### Maintaining and updating existing T cell epitope prediction tools

T cell epitope prediction tools are data-driven, meaning they are based on extracting patterns from experimental data. As more data become available over time, the tools can be retrained with the new knowledge to improve the accuracy of their predictions. This is particularly important for areas where little data is available to start with. For example, few improvements are expected at this point for prevalent HLA class I molecules where large numbers of data points are available characterizing the binding specificity, while significant gains are expected for e.g., HLA class II in general and HLA-DQ and DP molecules in particular where binding data are comparatively scarce. For both MHC class I and class II binding prediction tools, retraining is performed annually by (i) assembling the most current dataset from the IEDB, (ii) if enough new data points are available, retraining the algorithms based on this dataset, and (iii) updating the website to make the new algorithms available.

### Incorporating elution data in epitope prediction

Recent technical advancements in MS have led to the increased availability of large-scale datasets of peptides identified as naturally processed and presented by MHC molecules. These data now provide a valuable, complementary source of data in parallel to binding assays that can be used to improve MHC ligand prediction, as they provide an unbiased assessment of what peptides are found on the cell surface. Thus, they can give insights into the natural length distributions of presented peptides, which is impacted by processes other than MHC binding preference such as proteasomal cleavage, TAP transport, and ERAP Trimming (Trolle et al. 2016). Elution data can also reveal unconventional peptide-binding motifs. We and others could subsequently show that these unconventional motifs correspond to rare binding configurations with the peptide bulging out of the binding grove between anchors (Ebert et al. 2009) or being N-terminally (Pymm et al. 2017) or C-terminally (McMurtrey et al. 2016; Remesh et al. 2017) extended beyond the anchor position.

The advantages of elution data come with the fundamental disadvantage that detection of peptides by current methods is, at best, semi-quantitative and heavily impacted by factors such as intracellular protein expression and turn-over levels. In contrast, MHC binding assays provide quantitative affinities of defined peptides. We recently developed novel artificial neural network topology that allows the training of a single neural network on both binding and ligand elution data for MHC class I molecules simultaneously, thereby learning the commonalities and differences of the two types of data and boosting the overall predictive performance for epitope and MHC ligand identification (Jurtz et al. 2017).

### Immunogenicity predictions beyond MHC ligand binding and presentation

In a systematic comparison of immunogenic peptides known to trigger T cell responses with MHC affinity-matched peptides that did not (Calis et al. 2013), we found enrichment of certain amino acids, such as tryptophans, in the immunogenic peptides. This enrichment presumably reflects that certain amino acid side chains have an enhanced likelihood of being recognized by T cell receptors as non-self. We incorporated this into a prediction tool that provides a score, independent of MHC binding capacity, for how likely a peptide is to be immunogenic, which is now part of the IEDB toolset. We have recently extended this strategy using more complex machine learning approaches (such as neural networks), and on extending the same approaches to MHC class II restricted T cell recognition (Dhanda et al. 2018a).

### Integration of prediction steps into a single wizard

We found that many users can find it daunting to apply prediction tools in real-life applications for their work, especially if they do not have experience with bioinformatics tools. The problem is not that the tools themselves are difficult to run, but that users struggle to decide what to do with the output and simply adding documentation does not seem to overcome the problem. To address this, we have implemented TepiTool (Paul et al. 2016), which breaks the prediction process into small steps and, depending on choices (e.g. MHC class I vs. class II), changes what steps are necessary next. We have found that users were more likely to complete a prediction task using this tool compared with traditional prediction tools, where many offered steps have to instead be performed outside of the tool itself using custom scripts or advanced spreadsheet manipulations. Given its popularity, we intend to continue adding to the capabilities of TepiTool. In this context, we will also make available the tools EpiSelect (Perez et al. 2008), an algorithm that aims to select a given number of epitopes in a set of viral strains so that most strains are covered with the largest number of epitopes, and PopCover (Buggert et al. 2012), a method for selecting peptides with optimal population and pathogen coverage.

### B cell epitope prediction tools

The performance of present B cell epitope predictions is far behind that of T cell epitopes. Over the years, however, important technical advances have been made in producing mAb from human B cells and in sequencing antibodies, both of which are now becoming routine. Also the ability to simultaneously screen antibodies for reactivity against large-scale libraries promises to speed up epitope discovery. Taken together, these developments lead to the expectation that a much larger amount of well-characterized human antibody data will become available in the literature in the near future. This opens the possibility to improve tools either by the IEDB team or by outside scientists. Those tools need to be evaluated and, if found useful, implemented in the IEDB.

Until such new tools become available, we continue to maintain and update the present set of tools which comprise several classic amino acid propensity scale-based tools for which there previously was no web implementation available. In addition, we have developed and implemented several novel tools that represent state-of-the-art methods including, BepiPred (Jespersen et al. 2017; Larsen et al. 2006), DiscoTope (Haste Andersen et al. 2006; Kringelum et al. 2012), and ElliPro (Ponomarenko et al. 2008).

### Epitope prediction tools for specific BCR or TCR receptor sequences

The recent advent of specialized sequencing technologies for BCR/antibody and TCR sequences has greatly increased the number of adaptive immune receptors for which the epitope is known. The sequence of these receptors, especially the CDRs that are the most variable and that are in direct proximity to the antigen-binding site, is responsible for their epitope specificity. With increasing amounts of these data available, it will be feasible to unravel the rules that determine the specificity of these receptors, towards the ultimate goal of predicting, for a given antibody or TCR, what epitope and antigen it can recognize. We fully expect that this vision is a decade or so away from reality, but already tools are emerging that are taking steps towards this goal (Glanville et al. 2017; Jespersen et al. 2019; Ponomarenko et al. 2011; Sela-Culang et al. 2014).

Specifically, we have shown in the past that for a set of antibodies recognizing the same protein antigen, and for which there is cross-blocking data available (meaning it is known which antibodies do or do not bind in the same antigenic site), we can predict, with greatly increased accuracy, what antigenic sites are recognized on the antigen and which sets of antibodies recognize which site (Kim et al. 2009; Klausen et al. 2015; Peters et al. 2006). This approach takes full advantage of the fact that combining many weak predictors can lead to strong predictive outcomes. While any individual antibody:antigen recognition event can only be predicted with low accuracy, combining predictions for groups of antibodies and interrogating consistency with cross-blocking data achieves this goal.

Other approaches have recently shown that for TCRs, there is also a detectable complementarity of the CDR receptor sequences and the epitope recognized, allowing the target of a TCR to be predicted from its sequence. Again, applying consensus approaches that utilized data from donors and integrated HLA restriction patterns showed the most promise to identify epitopes (Glanville et al. 2017). Likewise, it has recently been shown that simple force-field-based methods combined with structural modeling of the TCR:p:MHC complex can be used to identify the cognate target of a TCR (Greenbaum et al. 2007).

We anticipate that this field will rapidly advance and different tools will emerge. We have so far implemented Lyra (Klausen et al. 2015), a tool that predicts the structure of TCR or antibody receptors based on their sequence. We are currently working on assembling datasets for epitope-specific antibodies and TCRs to analyze them for general patterns of complementarity with this now vastly larger dataset compared with our previous work, and to integrate them into an updated method of antibody- and TCR-specific epitope prediction. Even if accuracy of these tools will initially be low, providing them as a reference with curated benchmark datasets and standardized performance evaluation schemes will help advance the field.

### Benchmarking activities to determine the accuracy of prediction tools

Publicly available MHC class I and II binding predictions are routinely benchmarked by the IEDB team (Andreatta et al. 2017; Jensen et al. 2018; Kim et al. 2014; Kim et al. 2009; Peters et al. 2006; Trolle et al. 2015; Wang et al. 2008; Wang et al. 2010; Zhang et al. 2009), along with antibody epitope prediction tools (Kringelum et al. 2012; Ponomarenko and Bourne 2007). The results of these benchmark analyses and the accompanying datasets have proven highly valuable to the community, as evidenced by over 1800 citations to their corresponding publications. New prediction methods are continuously being developed by both the IEDB team and other groups, which makes continuous update of the benchmark results desirable. Such benchmarks inform tool developers on which approaches lead to the best prediction tools, and tool users can assess for themselves how individual tools perform. We therefore plan to continuously update our evaluations of the IEDB MHC binding prediction tools. The team will also make new benchmark datasets available for use by the tool development community. The evaluation process will also be expanded to include the T cell processing prediction tools, which were not previously evaluated.

For antibody prediction benchmarks, we previously have worked on community standards to determine how such a benchmark could be carried out (Greenbaum et al. 2007). In addition, we have assembled a carefully chosen set of non-homologous PDB structures to evaluate the expanding class of structure-based antibody prediction tools (Ponomarenko and Bourne 2007). While previous benchmarks have largely shown that existing antibody prediction tools have overall low performance (Blythe and Flower 2005), we are expecting that a number of new tools will be developed that benefit from the increase of high-quality data that is becoming available.

In addition to conducting performance benchmarks, we have also implemented an automated benchmarking system for prediction tools hosted inside and outside the IEDB, which is run on new datasets that become available in the IEDB before their release to the public. External developers are able to register their tools in these benchmarks and as new datasets become available, they are run through these tools immediately; allowing for side-by-side comparison of the predicted and experimental results using tools hosted in the IEDB. Overall performance metrics are reported and made available on a weekly basis. We have thus far implemented such benchmarks for MHC class I (Trolle et al. 2015) and MHC class II (Andreatta et al. 2017) binding predictions and plan to expand them to MHC ligand elution experiments (for both class I and class II), as well as T cell and B cell epitope recognition data, which will require additional consideration an input from tool developers.

## Community Outreach

The true value of the IEDB is best measured by the extent that it helps the user community of academic and applied scientists in their work. In this respect, the outreach and promotion efforts are crucial as they build awareness of the IEDB and keep the community apprised as the program evolves. Furthermore, the IEDB grows best when there is constant input from the scientific community, in particular, critical feedback on each of its key components, ranging from suggestions on recuration, query and reporting, the web portal interface, and the nature and utility of the tools provided in the Analysis Resource. From public launch to present day, the IEDB has been promoted in a variety of channels familiar to the scientific community, and these include publications in peer-reviewed journals and participation at conferences in the form of scientific presentations and exhibit booths.

### IEDB publications and meeting booths

As of December 2018, the IEDB team produced a total of 148 publications since program inception in 2003. Through publications of our work, we demonstrate that the various additions and revisions to components of the IEDB are actively and positively presented to the research community. Unlike advertisements, publications in peer-reviewed journals have ensured that the work performed was of scientific interest and met recognized quality standards in the immunology and bioinformatics communities.

To advertise the IEDB to potential users and interact with current users, we have utilized presentations and booths at scientific meetings. Between January 2012 and December 2018, the IEDB staff delivered 118 talks and posters at scientific meetings. This has been an important component of the outreach efforts as it has provided an opportunity to discuss the IEDB with prominent scientists and general users alike. In addition to scientific presentations, the IEDB has also hosted exhibit 29 booths at various meetings, selected to reach a wide breadth of user communities, ranging from scientists with expertise in basic immunology (AAI), infectious diseases (ASM Microbe), allergy (AAAAI), and autoimmune diseases (FOCIS). In our experience, booths have provided an opportunity for a more hands-on, informal, and in-depth interaction with prospective users. The booths allow staff to demonstrate queries and the use of tools on a one-on-one basis, a clear preference to some users. These interactions are also an alternative source of feedback. User comments are captured by IEDB personnel and are subsequently considered to identify new features and enhancements relevant to the user community. If agreeable, contact information of booth visitors is captured to allow for further interactions.

### Annual training/User Workshops

Since year 2012, the IEDB staff has prepared and run an annual IEDB User Workshop with the goal of providing hands-on training on searching for data in the IEDB and the use of the epitope prediction and analysis tools in the IEDB-AR. The program of this 2-day workshop is set up to explain the data structure, curation process, and the various ways to query the database on the first day, while the second day is devoted to review of the epitope prediction and analysis tools in the Analysis Resource. Both days include time for individual help. In 7 years of hosting this event, there have been 192 workshop participants and another 46 who participated in the live webcast. Additionally, a total of 56 travel fellowships were provided to facilitate participant attendance. Attendees range from graduate students and postdocs to government scientists and professors. We limit the number of on-site participants so we can provide personalized attention and address individual needs. Holding the workshops at free facilities also helps to minimize costs. Feedback from participants has been very positive and we have been able to gather a large number of recommendations from them on how to improve the IEDB, which is facilitated by multi-hour interactions at the workshops that are not normally available.

### Providing engaging and accessible help to users

Assistance to IEDB users is offered in two ways: an online help desk where users can ask for help with specific questions and a knowledgebase with articles and tutorials guiding users through a large collection of recurring help topics.

The IEDB help desk offers customized solutions to user questions. Users can access the help desk in several ways, including sending questions directly to help@iedb.org or from Help Request buttons which are incorporated at the top and bottom of each page on the database and analysis resource websites.

**Fig. 8:**
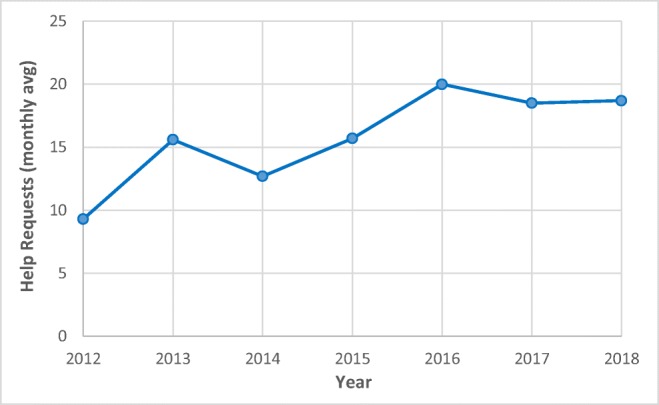
Average number of help requests to the IEDB Solutions Center per month

The help desk is part of the IEDB Solutions Center, which is hosted on Zendesk, a commercial web platform designed for handling help requests and developing a knowledgebase that users can access. For the contract period from 2012 to 2018, the IEDB received 1213 inquiries via the Solutions Center from users worldwide. In 2018, the IEDB averaged 18 help requests per month, doubling the number of requests received in 2012 (Fig. 8).

Upon submitting a help request, Zendesk provides an automated notice of receipt; however, the IEDB outreach team defines self-directed, response time goals each year. For example, in 2018, our goal was to have a qualified team member provide an initial response within one business day. Of the 224 total tickets received during 2018, initial responses were sent to 90.2% of requests within eight business hours, 25% of which were resolved outside of business hours. We also further aimed to resolve 85% of the general support requests within five business days. Of the 144 general support tickets received during 2018, resolution was achieved for 89.6% of requests within five business days, 17.8% of which were resolved outside of business hours. Most tickets that required more than two business days to resolve had insufficient information provided by the user in the initial report, which meant that further clarification was necessary for us to replicate the user’s problem. In other cases, the help requests involved standalone tools or one of the tool APIs that required input from the bioinformatics IT staff. Although an efficient system exists for reassigning support tickets, rerouting necessitates additional time.

To put our help desk service into perspective, we utilized the Zendesk reporting feature comparing our response and satisfaction times with “industry standards.” Once a ticket is solved, the user has the option of rating the IEDB’s response as Good or Bad through the Zendesk platform, which also tracks how quickly the ticket was resolved. For the last quarter of 2018, the IEDB had a satisfaction rating of 94% and an average first reply time of 4.66 h. The benchmark for companies of similar size was a 93% satisfaction rating and first reply in 20.5 h. The IEDB lies within the benchmark satisfaction rating and compares quite favorably in terms of response time to other companies.

### Establishing a knowledgebase with help links and tutorials

The IEDB Solutions Center Forum (http://help.iedb.org) that houses the knowledgebase can be accessed on the Help menu under Support in the banner or the Solutions Center link at the bottom of each page on the main website. It can also be accessed via the help link at the bottom of each page in the IEDB-AR. All documentation is placed in the Solutions Center, such as site release notes, the Annual Compendia, and publication announcements. There are also categories for frequently asked questions (FAQ), data submission, and the use of the analysis and prediction tools. A user can direct query the knowledgebase to find these articles, or instead may access these via redirect from numerous help links within the IEDB website, denoted with a question mark icon. The knowledge base also links users to webcast presentations taken from the annual User Workshop which can be used as “How-to” instructional references. All content of the knowledgebase is updated on an ongoing basis as the content of the IEDB evolves over time.

### Assessing the impact and general usage of the IEDB

**Fig. 9:**
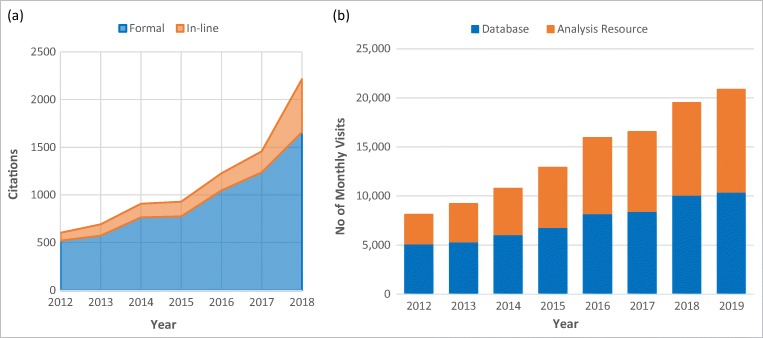
Impact of the IEDB assessed in terms of citations from published literature (**a**), and website usage based on median visits per month (**b**)

We periodically perform overall usage analysis, taking advantage of Google Analytics, which is a free web analytics service to track and report website traffic. Here we present an analysis of IEDB usage in recent years. As of July 2019, the main website has received over 10,300 visits per month from approximately 5400 unique visitors, while the IEDB-AR has received almost 10,400 visits per month from 4600 unique visitors. Figure 9b a demonstrates how the usage measured in median visits per month has increased since 2012 for both the main IEDB website and the Analysis Resource. This represents an increase of 102% for the former and a 249% increase for the latter. Based on interactions with IEDB tool users at workshops and conferences, we assume that one reason for the more rapid increase in usage of the IEDB-AR is that the epitope prediction tools are used by a wider community that includes researchers studying cancer and HIV, for whom the current IEDB database has limited utility as references in these respective domains are presently excluded from curation based on NIAID funding priorities.

The community of users is also international. In 2018, the geographic breakdown of users (measured by visits to the main website) included Asia (49%), the Americas (29%; including 22% USA), Europe (18%), Africa (3%), and Oceania (1%). For the Analysis Resource, the geographic breakdown included Asia (34%), Europe (31%), the Americas (29%; including 21% USA), Africa (3%), and Oceania (2%). These data illustrate the broad reach of the IEDB across the world.

During this same period of time, the main website received 46% of its traffic from organic searches, (i.e., visitors coming as a result of using a search engine). In addition, 44% of the traffic came from users directly accessing the website, and 10% came as a result of following a hyperlink from another website. Of the users arriving at the IEDB via site referral, approximately 23% of the referrals came from the links established on PubMed and 14% came from the Analysis Resource website. Similarly, in 2017, the IEDB-AR received 47% of its traffic from organic searches, 31% by referral from another website (86% of which were from the IEDB main website), and 22% from users going directly to the website.

The metrics described above measure overall usage, but do not provide information regarding how the website is navigated. To address this, we have expanded our use of Google Analytics and GoAccess. Google Analytics is implemented on each web page and helps us to identify traffic trends, such as direct links or contextual-based searches, both immediately and after modification of a page. These tools also help us to identify how many sites reference the IEDB, how well indexed the IEDB is, and how quickly each page is loading.

### Assessing the impact of the IEDB through publications

Another systematic metric illustrating the impact of the IEDB program on the scientific community is the number of citations made by authors in peer-reviewed journals. The citation analysis is conducted in the second quarter since experience has shown that there is a lag before all references of the preceding year are entered into PubMed, Google Scholar, and the ISI Web of Knowledge, the three sources used to find citations. In 2018, for instance, the IEDB received 2215 citations, continuing our streak of annual increases in received citations. We have also more recently found that as the popularity of the IEDB grows, there is often no actual citation provided but the IEDB is simply mentioned in the body of a document. To capture and monitor this type of informal citation, we introduced a formal analysis of the number of times the IEDB is referred to “in-line” as a resource (Fig. 9a). Using Publish or Perish, a software program that retrieves citations across Crossref, Google Scholar, Microsoft Academic, Scopus, and Web of Science, we reanalyzed all citations to the IEDB 2012–present and identified nearly 1500 in-line references to the IEDB which would have been overlooked during initial analysis since a specific IEDB-related publication was not cited.

Throughout the identification of citations, it is important to note that IEDB-authored articles citing other IEDB articles are specifically excluded to avoid artificially inflating the count.

Internally, citations identified during the annual analysis are further evaluated by subdividing them by publication year and category, such as retrieval of specific T or B cell datasets, utilization of specific tools, ontology development, or development of predictive or analytical tools. Inspection of this subdivided list allows for identification of trends, to highlight which areas and types of activities are most impactful and best received. Conversely, this analysis reveals areas where outreach could be improved to enhance IEDB relevance and impact.

## Conclusions

The primary focus of the IEDB initiative, which began in 2003, has been to ensure public accessibility to curated immunological data and bioinformatics tools; an objective which is achieved through continuous evolution of the behind-the-scenes systems infrastructure, the internal data processing tools, and established online web portals. Beyond basic existence, the IEDB strives to foster continued growth of the resource by iteratively updating its components, adding and improving features to make it more valuable to its user community. Adopting this proactive, community-oriented approach has enabled the IEDB to become a widely recognized and respected resource; evident through a user-base which has more than doubled in size and an annual citation count which has almost quadrupled since 2012. In order to maintain successful operation, the IEDB will rely on the day-to-day practices and procedures which have been refined over the past 16 years and will retain our over-arching vision to improve the acquisition, management, analysis, and dissemination of data and knowledge across the immunology research.
